# Tolerance to Dizziness Intensity Increases With Age in People With Chronic Dizziness

**DOI:** 10.3389/fneur.2022.934627

**Published:** 2022-07-14

**Authors:** Tino Prell, Sarah Mendorf, Hubertus Axer

**Affiliations:** ^1^Department of Geriatrics, Halle University Hospital, Halle, Germany; ^2^Department of Neurology, Jena University Hospital, Jena, Germany; ^3^Center for Vertigo and Dizziness, Jena University Hospital, Jena, Germany

**Keywords:** dizziness, vertigo, older age, depression, mediation

## Abstract

**Background:**

Dizziness is a common complaint in older adults. To know which factors are instrumental in enabling patients with chronic dizziness to tolerate their symptoms to a certain degree in everyday life can help to develop tailored therapies.

**Methods:**

Data from 358 patients with chronic dizziness and vertigo who had attended a multimodal daycare treatment program were recorded. Data included sociodemographic parameters, dizziness-related characteristics, the Vertigo Symptom Scale (VSS), and the Hospital Anxiety and Depression Scale (HADS). Descriptive statistics, elastic net regression, and mediation analysis were used.

**Results:**

A higher tolerance of dizziness was associated with higher age, higher intensity of dizziness, lower burden of dizziness, higher HADS depression, structural reason for dizziness (type), permanent dizziness, absence of attacks, and longer disease duration. In contrast, younger persons with attack-like dizziness reported to tolerate less dizziness. Age had a significant direct effect on tolerance (72% of the total effect) and a significant indirect effect *via* intensity on tolerance (28% of the total effect) in the mediation analysis.

**Conclusion:**

It can only be speculated that negative stereotypes about age-related complaints may play a role in this. Why older people tolerate more dizziness and to what extent this may contribute to lower healthcare utilization need to be investigated in further studies.

## Introduction

Vertigo and dizziness have a high lifetime prevalence and affect about 15% to over 20% of adults yearly in large population-based studies (1). They are caused by a variety of conditions and strongly influence activities of daily living and quality of life. Chronic or permanent dizziness often requires a multidisciplinary approach in specialized centers (2, 3) and goes along with high personal and healthcare burden (4). Especially, for chronic dizziness, it is essential that patients cope with the dizziness symptoms in daily living. In many chronic forms of dizziness, this means that patients have to tolerate dizziness to various degrees. The subjective perception of chronic dizziness is influenced by personality traits, psychosocial factors (e.g., anxiety with regard to unforeseeable recurrence), associated symptoms, and the course of disease (unpredictable attacks or permanent problems) (5). With regard to therapeutic approaches, it is crucial to know what factors influence the patient's ability to deal with chronic dizziness.

Every person has different desires and expectations that are usually different from their current state of living. In the context of quality of life, this difference or gap between the hopes/expectations and actual experiences of an individual is called the Calman gap (6). This gap mainly influences the quality of life and individual satisfaction. If the individual's current condition worsens in the context of a disease (e.g., chronic vertigo), then the gap between the current and desired state may increase, thereby lowering the satisfaction of the individual. On the other hand, individuals can adapt their expectations and desires (e.g., tolerate dizziness under some circumstances). Patients can have different understandings of their illness and other reference points, which can change over time or with regard to treatment options. In the case of chronic diseases, patients do not necessarily expect the complaints/symptoms to disappear completely as a result of treatment (6). Rather, patients are willing to tolerate some of the discomfort. In the context of this study, we were interested in understanding which factors are instrumental in enabling patients with chronic dizziness to tolerate their symptoms to a certain degree in everyday life. This should serve as the basis for future studies that aim to adapt therapy programs more individually (e.g., focus on personal resources and resilience). Therefore, our question was how much dizziness intensity can be tolerated by affected persons and what factors modulate this tolerance to dizziness?

Based on theoretical considerations and the existing literature, we considered the following parameters as relevant for our research question. First, we considered the individual intensity of dizziness and the burden due to dizziness as relevant cofactors. These are linked to the type of dizziness (attack vs. permanent) and the dizziness-associated handicap (7–9). Two patient-reported scales have been widely used to evaluate dizziness-associated handicap and severity in patients with vertigo: the Dizziness Handicap Inventory (DHI) and the Vertigo Symptom Scale (VSS) (10, 11). In contrast to the DHI, the VSS assesses the frequency of vestibular-balance symptoms and the severity of autonomic-anxiety symptoms, which have a great impact on quality of life. We therefore used the VSS to comprehensively describe dizziness. We also assumed that the vertigo diagnosis plays a role, in particular, with regard to the distinction between somatic vs. psychic reasons for chronic dizziness (7, 9). Dizziness symptoms are often accompanied and interact with psycho-physiological symptoms, especially depression and anxiety (11–14). Besides anxiety and depression, autonomic symptoms are also known to affect dizziness handicap (15). In addition, age and gender might influence how much dizziness a person is willing to tolerate. It is known from other studies that coping with distinct or multiple diseases is influenced by age and sex (16).

To answer our research question, we analyzed data from patients with chronic dizziness attending our multimodal treatment program in the Center for Vertigo and Dizziness, Jena, Germany (3).

## Methods

### Participants

This was a subgroup analysis of a larger longitudinal observational study in people with chronic dizziness attending a specialized vertigo center (17). The study was approved by the local ethics committee (Ethics Committee of the Friedrich-Schiller-University Jena, Number 5426-02/18), and written informed consent for study participation was obtained from all patients. The Center for Vertigo and Dizziness is a tertiary care outpatient project consisting of neurology, ear, nose, and throat and physiotherapy departments. The center provides interdisciplinary diagnoses of patients with chronic vertigo and dizziness and a day care multimodal therapy (2). Requirements for participation in this outpatient treatment program were chronic dizziness and vertigo (i.e., symptoms have persisted for at least 3 months or attacks have recurred at least 5 days per month), physical independence (i.e., walking independently), and no cognitive limitations that may affect the activities of daily living (3). During the study period (June 2013 to September 2017), 754 patients were treated in this therapy program. Six months after attendance of the therapy week, the patients were contacted *via* mail and asked to fill out questionnaires for follow-up assessment. Here, 444 (58.9%) of the patients completed the questionnaire and sent it back. Comparison between people with and without follow-up is given in Supplement Table S1. From these 444 datasets, 358 provided information about the dependent variable.

### Dependent Variable

The item “Now indicate what intensity of dizziness would be tolerable for you with successful treatment?” (German “Geben Sie jetzt an, welche Schwindelintensität für Sie bei erfolgreicher Behandlung erträglich wäre.”) was rated on a visual analog scale from 0 (no dizziness tolerable) to 10 (the strongest dizziness). This variable is termed “tolerance” (metric).

### Independent Variables

We assessed the following sociodemographic and vertigo- and dizziness-related data:

- Age (metric; years), gender (binominal; male/female).
- Vertigo diagnosis as defined at our center; advanced neurologists and vertigo specialists medically diagnosed and psychologically evaluated every patient. Diagnoses are generally based on the International Classification of Vestibular Disorders (ICVD) of the Bárány Society.
- Description of symptoms (binominal; the presence or absence of attacks, permanent dizziness/vertigo).
- Duration of symptoms (binominal, 3–6 months, >6 months).
- The intensity of vertigo/dizziness in the last 6 months was quantified using a visual analog scale from 0 (no intensity) to 10 (maximal intensity).
- Burden due to vertigo/dizziness in the last 6 months was quantified using a visual analog scale from 0 (no burden) to 10 (maximal burden; German: “Wie belastet waren Sie durch den Schwindel durchschnittlich in den letzten 6 Monaten?”).
- Hospital Anxiety and Depression Scale (HADS) to estimate depression (18).
- Vertigo Symptom Scale to quantify the symptoms of vertigo and dizziness (19) by vertigo-balance (VSS-V) and autonomic-anxiety (VSS-A) subscale.

### Statistics

All analyses were conducted using IBM SPSS Statistics (version 25), R (version 4.1.1), and Jamovi (Version 1.8.2). Values are the mean and standard deviation (SD) or numbers and percentages. Normal distribution was determined using the Shapiro–Wilk test. Missing data were treated according to the pairwise deletion process. Spearman correlations were used for correlation analyses between different variables. Correlation between subgroups was compared using Fisher's *z* (20). Elastic net regularization was performed to determine the predictors of the dependent variable (tolerance). Elastic net regularization leads to parsimonious models, which are easier to interpret. Variable selection is performed by shrinking the parameters toward zero and attenuating overfitting, a well-known problem when applying regression models with a large number of predictors. A 10-fold cross-validation was performed to choose the best model with the lowest mean cross-validated error. Within the elastic net algorithm, variables remain in the model if the prediction error averaged over the 10-fold cross-validation samples is reduced. Regression coefficients of the model with confidence intervals (CIs) were reported. Elastic net regularization was performed using the package *glmnet* in R (version 3.6.2). Finally, a simple mediation model as implemented in Jamovi was used to study the mediating effect of age on tolerance *via* intensity by using a 1.000 bootstrapping procedure. All statistical tests were applied two-sided at a significance level of 0.05.

## Results

**Figure 1:**
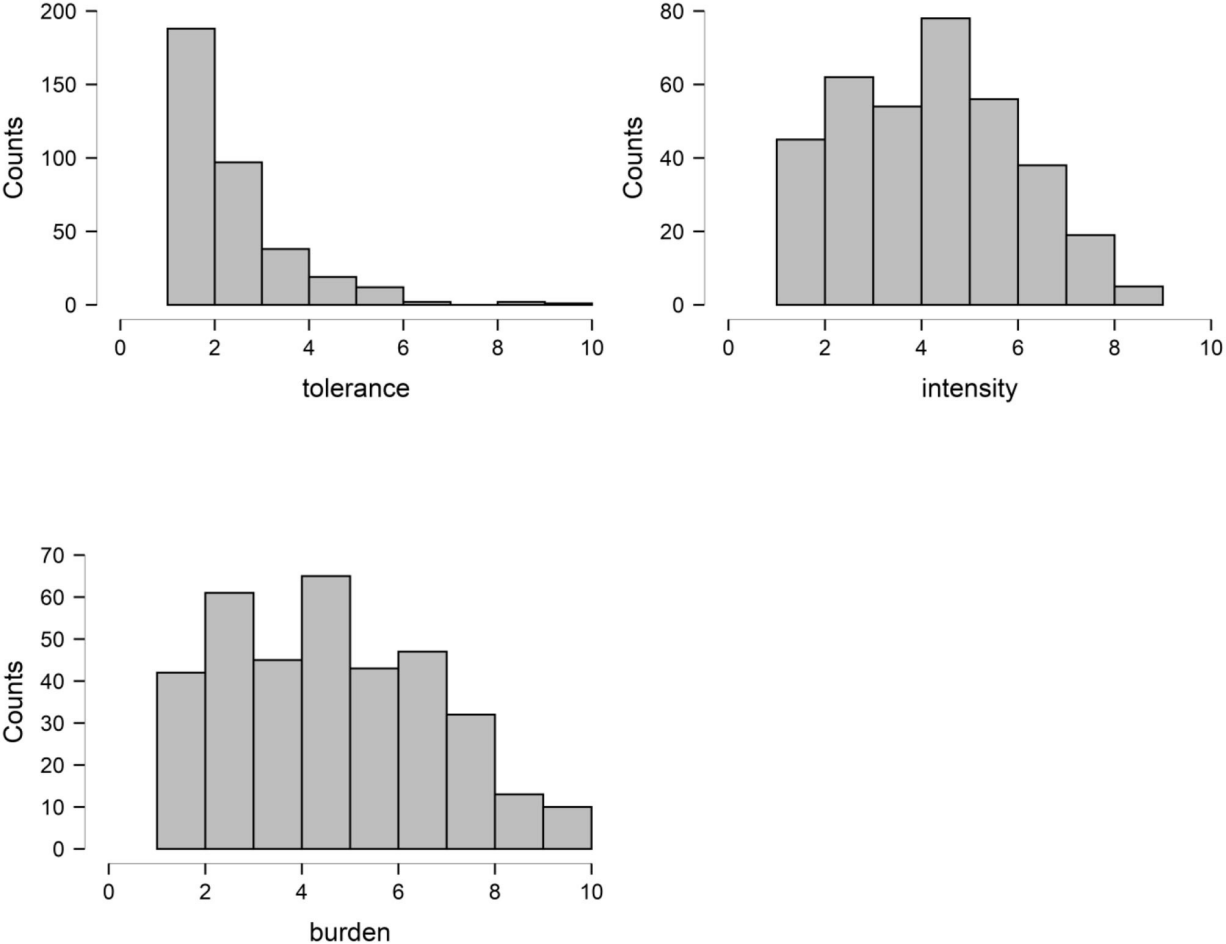
The distributions of intensity, burden, and tolerable dizziness intensity.

Sociodemographic parameters, diagnoses, and vertigo characteristics of the entire cohort are given in Table 1. The distributions of intensity, burden, and tolerance are displayed in Figure 1. The mean difference between the actual intensity of dizziness and the hypothetically tolerated dizziness (tolerance) was 2.0 (SD = 1.93) points on the visual analog scale. This means that patients wished that dizziness is reduced by ~20%.

**Table 1:** Overview of descriptive data.

		***M***	**SD**
**Age**		57.58	14.60
**VSS vertigo/balance subscale**		8.89	8.81
**VSS autonomic/anxiety subscale**		11.94	9.42
**VSS total**		20.82	16.16
**HADS depression**		5.53	3.86
**Tolerance (0–10)**		2.65	1.42
**Average intensity of dizziness (0–10)**		4.64	1.85
**Average burden of dizziness (0–10)**		5.04	2.21
		***n***	**%**
**Age group**	Younger (20–50)	113	31.6
	Middle (51–64)	124	34.6
	Older (>65)	121	33.8
**Sex**	Female	215	60.1
	Male	143	39.9
**Type**	Organic	199	56.9
	Psychic	126	36.0
	Unspecific	25	7.1
**Diagnosis**	BPPV	12	3.4
	BV	16	4.5
	CV	20	5.6
	MD	27	7.5
	MultD	64	17.9
	PPPD	156	43.6
	VM	13	3.6
	VN	36	10.1
	VP	6	1.7
	VS	8	2.2
**Permanent vertigo**	Yes	188	55.8
	No	149	44.2
**Attacks**	Yes	194	62.0
	No	119	38.0
**Duration of vertigo**	3–6 months	37	10.9
	>6 months	304	89.1

*HADS, Hospital Anxiety and Depression Scale; VSS, Vertigo Symptom Scale; BPPV, benign paroxysmal positional vertigo; BV, bilateral vestibulopathy; CV, central vertigo; MD, Meniere's disease; MultD, multisensory deficit; VM, vestibular migraine; VN, vestibular neuritis; VP, vestibular paroxysmia; VS, vestibular schwannoma*.

**Figure 2:**
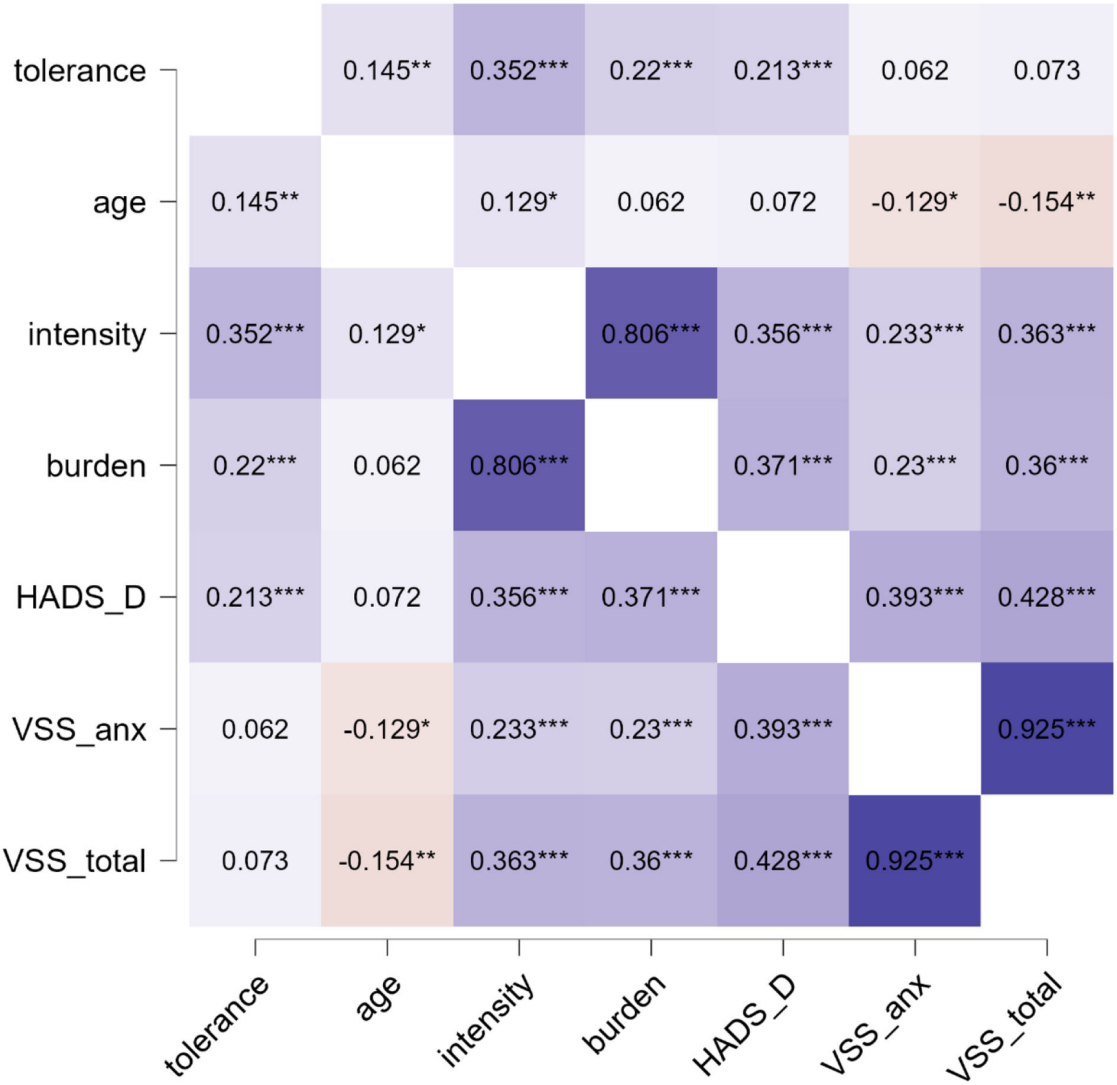
Spearman's rho heatmap. *p < 0.05, **p < 0.01, ***p < 0.001. Blue indicates positive and red indicated negative correlation. HADS, hospital anxiety and depression scale; VSS_anx, vertigo severity scale autonomic-anxiety; VSS_total, total vertigo severity scale.

**Figure 3:**
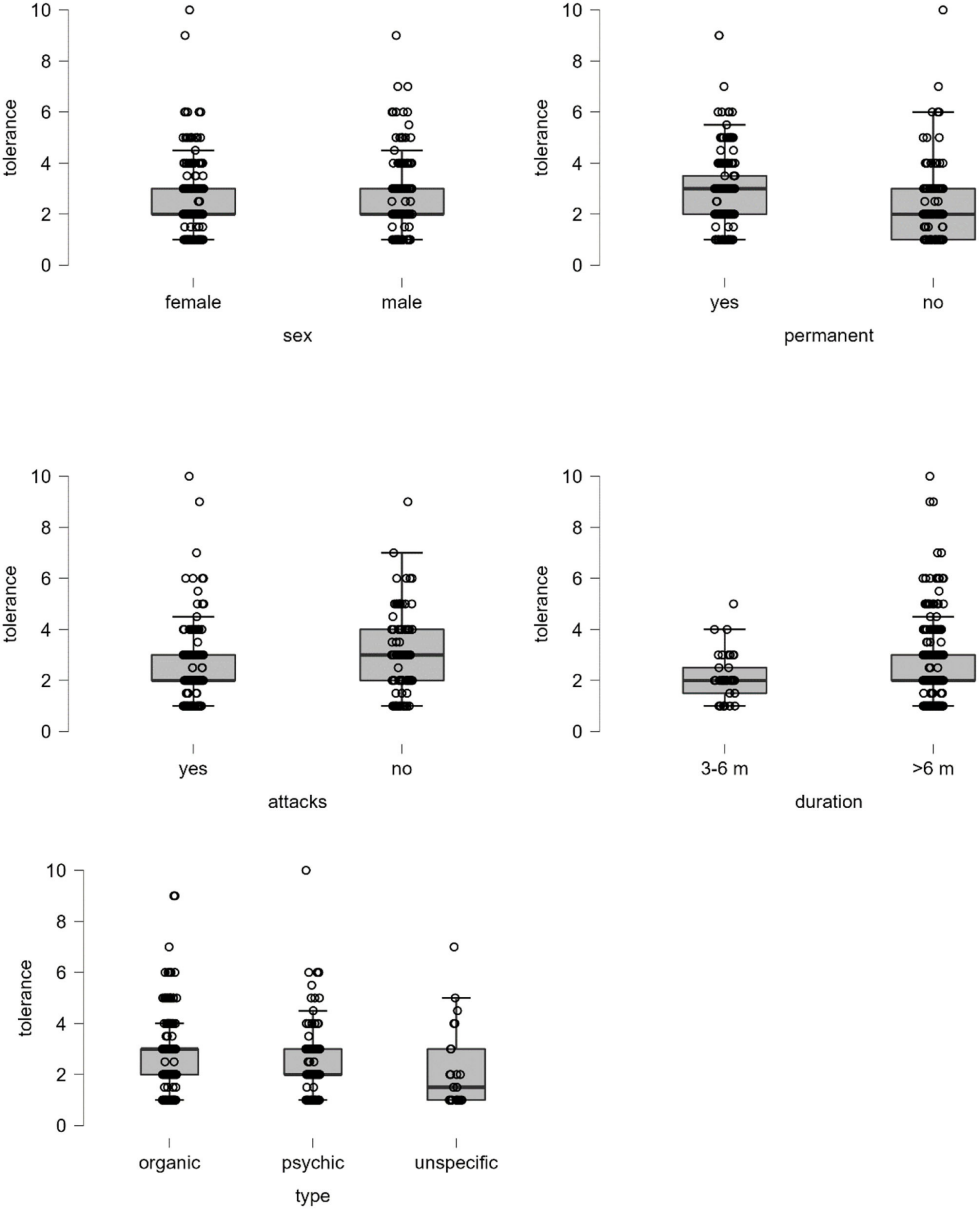
Boxplots for tolerance. Test statistics for Mann–Whitney *U*-test: sex *p* = 0.632, permanent *p* < 0.001, attack *p* < 0.001, and duration *p* = 0.026. Test statistic for Kruskal–Wallis test: type *p* = 0.001, unspecific – psychic *p* = 0.075, unspecific – organic *p* = 0.002, psychic – organic *p* = 0.016.

Spearman correlation between tolerance and independent variables is displayed in Figure 2. Here, tolerance correlated with age, the intensity of dizziness, the burden of dizziness, and HADS depression. In the non-parametric tests, significant group differences for tolerance were found in dizziness type (higher tolerance for organic reasons in comparison to psychic or unspecific vertigo), the permanence of vertigo (higher tolerance in permanent dizziness), attack-like dizziness (lower tolerance for attacks), and duration of dizziness (higher tolerance for longer disease duration), but not for sex (*p* = 0.63; Figure 3).

The significant parameters from univariate analyses were then entered into an elastic-net model. A higher tolerance of dizziness was associated with higher age, higher intensity of dizziness, lower burden of dizziness, higher HADS depression, organic reason for dizziness (type), permanent dizziness, absence of attacks, and longer disease duration (Table 2).

**Table 2:** Elastic net regularization with tolerance as a dependent variable.

	**Coefficient**	**2.5% CI**	**97.5% CI**	***p*-value**
Constant	1.327	0.527	2.128	0.001
Age	0.002	−0.011	0.014	0.805
Intensity	0.325	0.186	0.465	0.000
Burden	−0.160	−0.275	−0.044	0.007
HADS-D	0.042	−0.003	0.086	0.069
Type (organic/structural)	0.182	−0.207	0.572	0.360
Type (unspecific)	−0.226	−0.903	0.451	0.514
Permanent (no)	−0.132	−0.509	0.246	0.495
Attacks (no)	0.296	−0.078	0.670	0.122
Duration (>6 months)	0.136	−0.394	0.666	0.616

*Model fit: χ^2^(9) = 91.118, p < 0.001, Pseudo-R^2^ (Cragg-Uhler) = 0.164, AIC (Akaike Information Criterion) = 979.879, BIC (Bayesian information criterion) = 1,020.057. Hospital Anxiety and Depression Scale (HADS)*.

**Figure 4:**
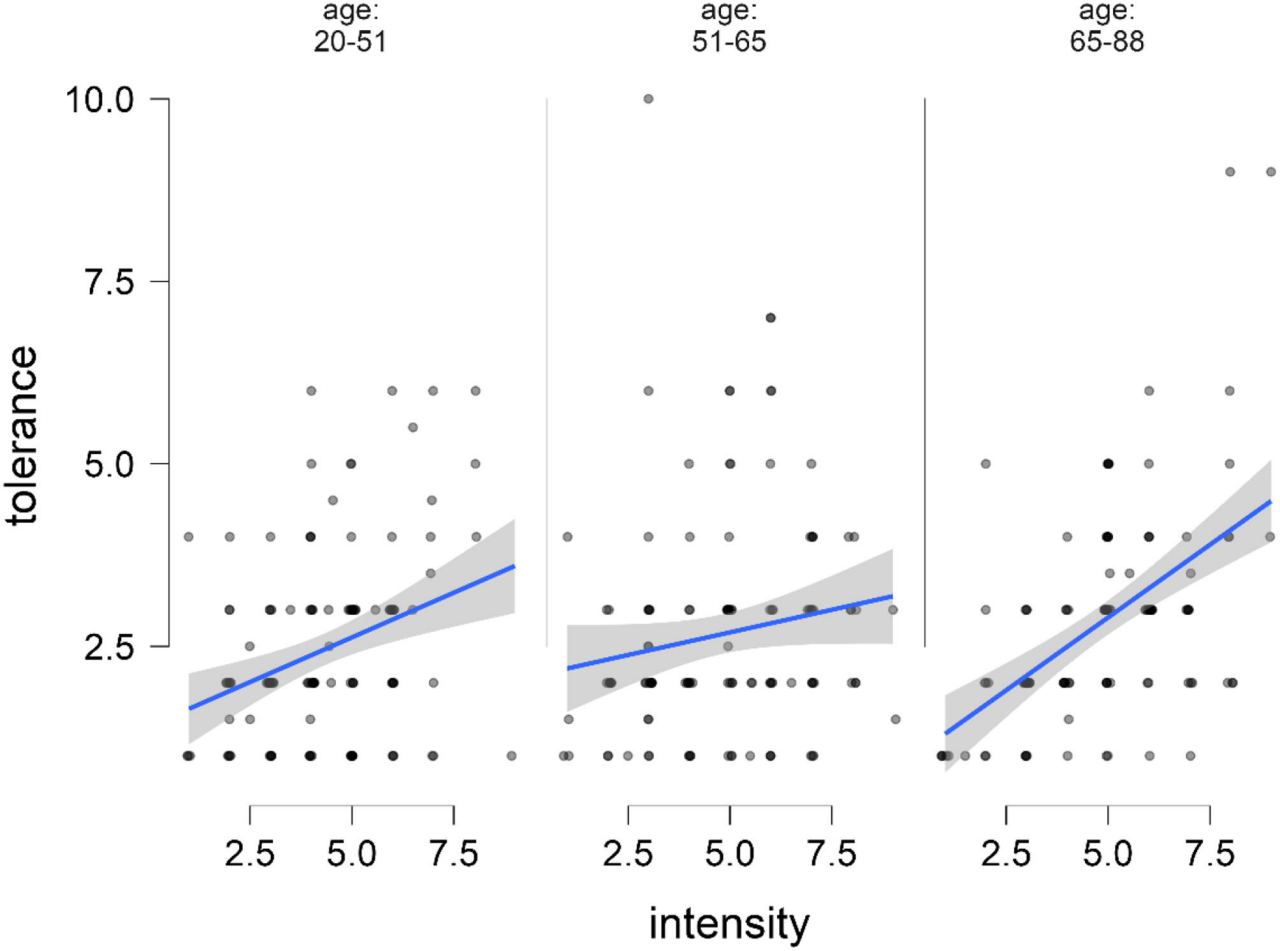
Flexplot.

The flexplot in Figure 4 shows that the correlation between tolerance and intensity differed between younger (*r* = 0.28, *p* = 0.003), middle-aged (*r* = 0.226, *p* = 0.012), and older persons (*r* = 0.50, *p* < 0.001). This means that higher intensity of dizziness is related to higher tolerance and this relationship is strongest in older adults (Fisher's *z* = −1.9780, *p* = 0.048 for young vs. older; Fisher's *z* = −2.3824, *p* = 0.017 for middle-aged vs. older). Given that age is also weakly correlated with intensity (Figure 2), we finally tested if the effect of age on tolerance could be mediated by intensity. Mediation analysis with 1.000 bootstrapping revealed that age had a significant direct effect on tolerance (72% of the total effect) and a significant indirect effect *via* intensity on tolerance (28% of the total effect; Table 3). Therefore, the effect of age on tolerance occurs mainly independent of the perceived influence.

**Table 3:** Mediation model.

**Effect**	**Estimate**	**SE**	**95% C.I. (a)**		**β**	***z***	***p*-value**	**% Mediation**
			**Lower**	**Upper**				
**Indirect effect**								
Age⇒intensity⇒tolerance	0.003819	0.001863	2.272e-4	0.007529	0.03891	2.050	0.040	28.12
**Component**								
Age ⇒intensity	0.015462	0.006882	0.002280	0.029258	0.12151	2.247	0.025	
Intensity⇒tolerance	0.246988	0.042355	0.162887	0.328915	0.32021	5.831	<0.001	
**Direct effect**								
Age ⇒ tolerance	0.009761	0.004457	0.001065	0.018536	0.09946	2.190	0.029	71.88
**Total effect**								
Age ⇒ tolerance	0.013580	0.005152	0.003483	0.023677	0.13837	2.636	0.008	100.00

*Confidence intervals (CIs) computed with method: parametric bootstrap. Betas are completely standardized effect sizes*.

## Discussion

Dizziness and vertigo are common complaints associated with a substantial individual burden (4, 21–23). It is important to understand the impact of dizziness on individual wellbeing (24). In this study, we investigated which factors influence the ability of patients with chronic dizziness to tolerate their dizziness symptoms in everyday life. Our independent variables included age, sex, common dizziness-related measures, and psychological parameters. In terms of dizziness-related measures, we found that dizziness can be better tolerated when a higher baseline intensity of dizziness is present, when the burden is low, when there is a structural reason for dizziness (type), and when the disease duration is longer. In addition, permanent dizziness is better tolerated than attack-like dizziness. Moreover, we found that dizziness is better tolerated in higher age and people with depressive symptoms. The strongest influence on tolerance has the intensity and burden of dizziness.

When patients were asked how much dizziness was tolerable for them, they indicated on average that 20% less dizziness would be desirable. Only a small proportion stated that dizziness was not tolerable at all (i.e., rated 0 on the visual analog scale). This difference is comparable to the Calman gap, i.e., the difference between the current quality of life and desired quality of life in people with chronic diseases (6). We acknowledge that there are different methods (e.g., Pictorial Representation of Illness and Self Measure) to measure the burden of dizziness (24). However, here we used simple visual analog scales to measure intensity, burden, and tolerance to dizziness in order to get comparable outcome parameters.

The intensity of dizziness/vertigo, age, depression, type of vertigo (somatic vs. non-somatic), and persistence of symptoms (permanent or attack-like) play a role in how much dizziness/vertigo is tolerable. Patients tolerate more dizziness/vertigo with increasing age. This could be demonstrated in the elastic net model after correction for cofactors and in the flexplot (Figure 4). This effect occurred independent from the intensity of dizziness/vertigo, although it is known that dizziness-associated handicap increases with age (25) and older patients with dizziness have less anxiety than younger patients (26).

In an earlier study, we found that younger people were more frequently hospitalized due to dizziness than older adults, and the number of consultations of healthcare providers was considerably higher in younger patients (26). Now, we can hypothesize that a higher tolerance of dizziness increases the threshold to initiate specialized vertigo therapy and could partly explain lower healthcare utilization in older patients. However, why older people tolerate more dizziness and to what extent this may contribute to lower healthcare utilization need to be investigated in further studies. At this point, it can only be speculated that also negative stereotypes about age-related complaints may play a role in this. Many studies provided evidence that ageism affects health outcomes (27–29). For example, when older adults are randomly assigned to a negative-age-stereotype condition, it impairs performance in cognitive tasks (30, 31). However, also age stereotypes that are directed at oneself in old age (self-perceptions of aging) affect functional health (29, 32, 33). Moreover, age stereotypes tend to be resistant to even extremely stressful events (34).

Another influencing factor for higher tolerance may be the finding that anxiety (measured with the VSS) does not play such a prominent role in the older patients with dizziness than in younger patients (3). Instead, we found that depressive symptoms (higher HADS-D) correlate with tolerance. To understand this finding, one has to keep in mind that depressive symptoms also correlate with the current intensity of dizziness. This means that people with depressive symptoms in general rate their current and tolerable dizziness higher in comparison to people with a lower HADS-D (while the gap between both remains stable). Given that older people with chronic dizziness report lower levels of depression and anxiety (26), we assume that the higher tolerance with increasing age occurs independently of these psychological cofactors. Our findings are in line with earlier studies, showing that depression is frequent and clinically relevant in people with dizziness and that depression influences the severity and outcome of dizziness (12, 35–37).

Our study has several limitations. Patients in our sample attended a highly specialized treatment program for chronic dizziness. Thus, there is a selection bias regarding their underlying diagnoses and the findings should be generalized with caution. For example, only a few people had benign paroxysmal positional vertigo (BPPV) in our cohort, a common cause of dizziness among older adults (38). This is because many patients with BPPV were already treated by their general physicians, which makes an appointment in our center not necessary. Patients are referred to our tertiary care center only if symptoms become chronic. Although the response rate to our survey was relatively good for a postal/mail survey, this also limits the generalizability of the results. Our dependent variable was so far not validated. Nevertheless, we assume that a visual analog scale is suitable to track differences in the individual perceptions of illness. It is worth to note that other methods also exist to determine the burden of dizziness (i.e., Pictorial Representation of Illness and Self-Measure) (24) and that the kind of assessment may influence the results. Finally, our analysis is driven by a theoretical selection of independent variables. Further studies are therefore needed to gain a deeper understanding of the relationship between, for example, age and tolerance of dizziness.

The ability to cope and tolerate dizziness depends on the type and presentation of vertigo, age, and psychological comorbidities. Looking at how much dizziness patients can tolerate in order to cope with everyday life could be an interesting approach to develop new therapies. At least this view enriches the mere consideration of the handicap caused by the dizziness. In further studies, our approach could be used to focus more on the resources and resilience of patients in order to strengthen them and thus to cope better with dizziness in everyday life.

## Data Availability Statement

The raw data supporting the conclusions of this article will be made available by the authors, without undue reservation.

## Ethics Statement

The studies involving human participants were reviewed and approved by Ethics Committee of the Friedrich-Schiller-University Jena, Number 5426-02/18. The patients/participants provided their written informed consent to participate in this study.

## Author Contributions

TP: design of the study and writing of the paper and analysis. HA: collection of data. HA and SM: revision of the paper. All authors contributed to the article and approved the submitted version.

## Conflict of Interest

The authors declare that the research was conducted in the absence of any commercial or financial relationships that could be construed as a potential conflict of interest.

## Publisher's Note

All claims expressed in this article are solely those of the authors and do not necessarily represent those of their affiliated organizations, or those of the publisher, the editors and the reviewers. Any product that may be evaluated in this article, or claim that may be made by its manufacturer, is not guaranteed or endorsed by the publisher.
