# Supplementary material for: Tolerance to Dizziness Intensity Increases With Age in People With Chronic Dizziness
Source: Front Neurol. 2022 Jul 14;13:934627. doi: 10.3389/fneur.2022.934627 (PMC9330066; doi:10.3389/fneur.2022.934627)
Supplement: Supplementary file 1 [file Data_Sheet_1.docx]

Supplement

|  | | **Patients without follow-up data** | | **Patients with follow-up data** | |  |
| --- | --- | --- | --- | --- | --- | --- |
| **Categorical variables** | | **n** | **%** | **N** | **%** | **p** |
| sex | Female | 246 | 62.8 | 215 | 60.1 | 0.448 |
|  | Male | 146 | 37.2 | 143 | 39.9 |  |
| diagnosis category | Somatic | 151 | 50.3 | 199 | 56.9 | 0.210 |
|  | Non- somatic psychogenic | 128 | 42.7 | 126 | 36.0 |  |
|  | Non-somatic unspecific | 21 | 7.0 | 25 | 7.1 |  |
| duration of symptoms | 3-6 months | 58 | 15.5 | 37 | 10.9 | 0.069 |
|  | >6 months | 317 | 84.5 | 304 | 89.1 |  |
| diagnosis | BPPV | 12 | 3.0 | 12 | 3.4 | 0.843 |
|  | BV | 19 | 4.8 | 16 | 4.5 |  |
|  | CV | 23 | 5.8 | 20 | 5.6 |  |
|  | MD | 28 | 7.1 | 27 | 7.5 |  |
|  | MultD | 53 | 13.4 | 64 | 17.9 |  |
|  | PPPD | 195 | 49.2 | 156 | 43.6 |  |
|  | VM | 17 | 4.3 | 13 | 3.6 |  |
|  | VN | 37 | 9.3 | 36 | 10.1 |  |
|  | VP | 4 | 1.0 | 6 | 1.7 |  |
|  | VS | 8 | 2.0 | 8 | 2.2 |  |
| permanent | Yes | 169 | 23.9 | 230 | 32.5 | 0.740 |
| dizziness | No | 121 | 17.1 | 187 | 26.4 |  |
| attack-like | Yes | 165 | 24.8 | 237 | 35.6 | 0.447 |
|  | No | 110 | 16.5 | 153 | 23.0 |  |
| **Metric variables** | | **mean** | **SD** | **mean** | **SD** | **p** |
| age (years) | | 57.74 | 15.34 | 57.58 | 14.60 | 0.439 |
| VSS-Vertigo-balance | | 11.15 | 8.99 | 11.27 | 8.38 | 0.423 |
| VSS-Autonomic-anxiety | | 14.66 | 10.65 | 13.23 | 9.89 | 0.062 |
| VSS total score | | 25.80 | 16.89 | 24.51 | 15.58 | 0.280 |
| HADS depression | | 6.32 | 3.83 | 6.03 | 3.96 | 0.301 |
| Abbreviations: BPPV, benign paroxysmal positional vertigo; BV, bilateral vestibulopathy; CV,central vertigo; HADS, Hospital Anxiety and Depression Scale; MD, Meniere's disease; MultD, multisensory deficit; PPPD, persistent postural-perceptual dizziness; VM, vestibular migraine; VN, vestibular neuritis; VP, vestibular paroxysmia; VS, vestibular schwannoma; VSS, Vertigo Severity Scale. | | | | | | |

**Supplementary Table S1.** Comparison between people with and without follow-up.

**Supplementary Tables S2.** Median and mean of tolerance for different group comparisons.

|  | | **tolerance** | | | | | |
| --- | --- | --- | --- | --- | --- | --- | --- |
|  | | **Age**  **20 - 51** | | **Age**  **52 - 65** | | **Age**  **> 65** | |
| Valid |  | 2.00 |  | 2.00 |  | 1.45 |  |
| Mean |  | 2.456 |  | 2.665 |  | 2.832 |  |
| Std. Deviation |  | 1.280 |  | 1.520 |  | 1.454 |  |
| IQR |  | 1.500 |  | 1.000 |  | 1.500 |  |
|  | | | | | | | |

|  | | **tolerance** | | | | | | |
| --- | --- | --- | --- | --- | --- | --- | --- | --- |
|  | | **organic** | | | **psychic** | | **unspecific** | |
| Median |  | 3.000 | |  | 2.000 |  | 1.500 |  |
| Mean |  | 2.812 | |  | 2.484 |  | 2.140 |  |
| Std. Deviation |  | 1.416 | |  | 1.361 |  | 1.611 |  |
| IQR |  | 1.000 | |  | 1.000 |  | 2.000 |  |
|  | | | | | | | | |
|  | | | | | | | | |
|  | | | | | |  |  |  |
|  | | **tolerance** | | | |  |  |  |
|  | | **female** | | **male** | |  |  |  |
| Median |  | 2.000 |  | 2.000 |  |  |  |  |
| Mean |  | 2.600 |  | 2.713 |  |  |  |  |
| Std. Deviation |  | 1.370 |  | 1.504 |  |  |  |  |
| IQR |  | 1.000 |  | 1.000 |  |  |  |  |
|  | | | | | |  |  |  |

|  | | | | | |
| --- | --- | --- | --- | --- | --- |
|  | | **tolerance** | | | |
|  | | **permanent** | | **not permanent** | |
| Median |  | 3.000 |  | 2.000 |  |
| Mean |  | 2.851 |  | 2.315 |  |
| Std. Deviation |  | 1.453 |  | 1.333 |  |
| IQR |  | 1.500 |  | 2.000 |  |
|  | | | | | |
|  | | | | | |
|  | | | | | |
|  | | **tolerance** | | | |
|  | | **attack-like** | | **no attacks** | |
| Median |  | 2.000 |  | 3.000 |  |
| Mean |  | 2.433 |  | 2.950 |  |
| Std. Deviation |  | 1.376 |  | 1.437 |  |
| IQR |  | 1.000 |  | 2.000 |  |
|  | | | | | |
|  | | | | | |

|  | | | | |  |
| --- | --- | --- | --- | --- | --- |
|  | | **tolerance** | | |  |
|  | | **3-6 months** | | **>6 months** |  |
|  |  |  |  |  |  |
|  |  |  |  |  |  |
| Median |  | 2.000 |  | 2.000 |  |
| Mean |  | 2.135 |  | 2.691 |  |
| Std. Deviation |  | 0.933 |  | 1.462 |  |
| IQR |  | 1.000 |  | 1.000 |  |
|  | | | | |  |
|  | | | | |  |
